# Perioperative Multimodal Anesthesia Using Regional Techniques in the Aging Surgical Patient

**DOI:** 10.1155/2014/902174

**Published:** 2014-01-20

**Authors:** Diana Nordquist, Thomas M. Halaszynski

**Affiliations:** Department of Anesthesiology, Yale University School of Medicine, Yale-New Haven Hospital, 333 Cedar Street, P.O. Box 208051, New Haven, CT 06520-8051, USA

## Abstract

*Background*. Elderly patients have unique age-related comorbidities that may lead to an increase in postoperative complications involving neurological, pulmonary, cardiac, and endocrine systems. There has been an increase in the number of elderly patients undergoing surgery as this portion of the population is increasing in numbers. Despite advances in perioperative anesthesia and analgesia along with improved delivery systems, monotherapy with opioids continues to be the mainstay for treatment of postop pain. Reliance on only opioids can oftentimes lead to inadequate pain control or increase in the incidence of adverse events. Multimodal analgesia incorporating regional anesthesia is a promising alternative that may reduce needs for high doses and dependence on opioids along with any potential associated adverse effects. *Methods*. The following databases were searched for relevant published trials: Cochrane Central Register of Controlled Trials and PubMed. Textbooks and meeting supplements were also utilized. The authors assessed trial quality and extracted data. *Conclusions*. Multimodal drug therapy and perioperative regional techniques can be very effective to perioperative pain management in the elderly. Regional anesthesia as part of multimodal perioperative treatment can often reduce postoperative neurological, pulmonary, cardiac, and endocrine complications. Regional anesthesia/analgesia has not been proven to improve long-term morbidity but does benefit immediate postoperative pain control. In addition, multimodal drug therapy utilizes a variety of nonopioid analgesic medications in order to minimize dosages and adverse effects from opioids while maximizing analgesic effect and benefit.

## 1. Introduction

The complex and multifactorial processes of aging can encompass all human organ systems. Secondary to cumulative effect(s) of comorbid condition(s) and diminished physiologic reserve, perioperative stresses can interfere with physiologic homeostasis and lead to potential deleterious adverse effects (AE). With an increase in number of elderly patients undergoing surgery, it is important to determine optimal perioperative therapies to improve recovery while minimizing AE for older surgical patients. A contribution to development of perioperative complications for patients is improper/inadequate postoperative pain therapy. Inadequate postsurgical pain management can be associated with poor interventional outcomes with potential higher rate(s) of medical complications, decreased perioperative pain anesthesia/analgesia experience for patients, and patient and family member dissatisfaction associated with their surgical encounter [1, 2].

Planning an anesthetic technique and perioperative pain regimen requires consideration of several interventional details. However, debate continues over the optimal approach to anesthesia and analgesia in the elderly. It is always important to consider patient age, preexisting comorbidities, anticipated surgical procedure, and postoperative analgesic requirements when deciding upon appropriate perioperative pain management strategies for older patients. Perioperative pain management methods examined and explored in this paper are regional anesthesia, including neuraxial and peripheral nerve blockade, along with multimodal drug therapy.

Older patients have age-related physiological and pharmacological differences that are unique. In addition, many geriatric patients suffer from poor perioperative health status and comorbid disease, varying degrees of physical de-conditioning (especially prior to lower extremity orthopedic procedures), and compromised organ reserve capacity. Cardiovascular, nervous, pulmonary, endocrine, and immune systems can all be affected by human aging processes. Systemic changes that accompany aging are carefully reviewed in order to determine some suggested optimal techniques for perioperative pain management.

The number and extent of any coexisting disease(s) and medical condition(s) are more directly related to perioperative risk than to chronological age for elderly patients. Therefore, patient age alone should no longer be considered a major risk factor for anesthesia and surgery. More important factors and better predictors for the elderly include issues such as overall physical status, medical history, and disease state along with patient condition (e.g., physical deconditioning) and type of surgery. Complication rates for perioperative anesthetic and pain management choices increase very little with the advancing age in absence of coexisting disease. However, there is often an increased incidence of disease and existing comorbidity(s) in older surgical patients. Four-fifths of older patients have at least one complicating medical condition and 1/3 have 3 or more coexisting diseases. There are also several medical conditions that are predictive of higher surgical risk such as hypertension, diabetes mellitus, and ischemic heart disease [3], and other predictors of increased perioperative risk in elderly patients include urgency, type, and duration of the planned surgery. Upper abdominal surgical procedures followed by thoracic and open-heart surgery are associated with the highest morbidity and mortality for older surgical patients.

## 2. Regional Anesthesia

There are several types of regional modalities available (Table 1) and investigations in regional anesthesia (RA; both neuraxial and peripheral nerve blockade) are variable and include different regional techniques combined with various drug regimens including (a) peripheral nerve blockade alone; (b) peripheral nerve blocks combined with general anesthesia; (c) neuraxial blockade; (d) neuraxial blockade with general anesthesia; and (e) neuraxial combined with peripheral nerve blockade. RA involves loss of sensory and/or motor function of a specific portion or dermatome of the body by temporary interruption of normal nerve conduction. Lack of consistency within RA studies and protocols remains an important factor that has limited the ability to portray firm indications, guidelines, and recommendations about any advantageous or optimal anesthetic technique in the geriatric population. For this reason, several retrospective and prospective studies fail to show any difference or meaningful objective outcome between general versus regional anesthesia in older patients. In addition, no significant long-term variation in morbidity or mortality has been established except evidence of a reduced incidence of deep vein thrombosis and reduced blood loss when RA is utilized [4].

Clinically, RA appears to be a very effective and safe technique in elderly surgical patients. Several established and theoretical benefits of RA are listed in Table 2. And even though RA has many advantages, there are also issues specific to the elderly that should be considered when choosing an optimal procedure-specific anesthetic/analgesic modality. For example, spinal anesthesia in older patients can have a reduced latency time likely due to diminished cerebral spinal fluid (CSF) and higher dermatome level achieved than in younger patients (due to spinal stenosis), along with increased blockade density. Reduced nerve myelination also results in a greater diffusion of local anesthetic and wider nerve block extension in the elderly; therefore, a reduced dosage of local anesthetic is recommended [4]. In order to provide high-quality care for older surgical patients, there should be a careful consideration of the patient's medical history, age-related physiologic changes, and anticipated altered effects from any chosen anesthetic technique.

## 3. Multimodal Approach to Pain Medicine in the Elderly

For a host of reasons and despite advances made in analgesia and newer analgesic techniques, monotherapy with opioids remains the mainstay of treatment for perioperative pain. Opioids (class of central-acting analgesics) provide powerful dose-dependent relief of pain. Opioids are the most commonly used medication for postoperative pain delivering good analgesic effect. Unfortunately, opioids can alter central nervous system (CNS) activity and lead to physical and psychological dependence with longer-term use, especially in the elderly. They may also be burdened with several potential adverse effects such as nausea, sedation, constipation, ileus, dysphoria, respiratory depression, and risk of abuse [5]. These opioid related adverse effects could be exacerbated due to altered pharmacokinetics and pharmacodynamics from age-related physiologic changes in the elderly. Some examples of pharmacokinetic changes can include reduced hepatic and renal perfusion along with a reduction in total body water that can alter clinical responses to these drugs in older patients. In addition, examples of pharmacodynamic changes can include an increased sensitivity to CNS depressant agents resulting in cognitive dysfunction and/or reduction in minimum alveolar concentration.

With dependence on opioid monotherapy, results may reveal inadequate postoperative pain management and can often be of limited value due to associated AE's. Opioid mono-therapy can lead to requirements of higher doses to achieve adequate pain control and with higher dosages, more side effects become possible as well as intensity of AE that often prove to be dose dependent. In the very recent past, use of multimodal drug therapy has gained value and utility as being a more effective strategy in perioperative pain control for all patients [6]. Multimodal analgesia may serve to optimize efficacy by using different analgesic drug classes, each of which uses different receptors and pathways for clinical effect(s) and improved surgical outcome.

Considering the aspect of less dependence on one medication treatment modality often provides the practitioner with an ability to use lower drug dosage of individual analgesics along with less reliance on one specific agent so as to limit AE from any single medication as well as possibly reducing tolerance and dependence. Physicians find this approach to perioperative pain management beneficial, as it can often permit less reliance on only opioids. Multimodal analgesia incorporating RA may also enhance recovery while enabling optimal rehabilitation, quicker transfer to an outpatient setting and more expeditious return to activities of daily living following major surgery. RA as part of multimodal analgesia may in turn reduce use of healthcare resources, help to minimize cost and provide value to end users of procedure-specific pain management treatment modalities. Models of multimodal analgesia can be designed to include fast onset of action, reduced AE's, tailored toward improved safety profiles in certain patient populations as well as effective reduction in pain levels by medication and analgesic delivery directed from multiple routes by different receptor interfaces. RA as part of multimodal anesthesia has been shown to be efficacious in multiple studies and may also improve outcome by reducing opioid use for optimal pain control in a wide variety of surgical interventions. In addition, since surgical patients rarely present with pure nociceptive or neuropathic pain issues (typically a mixed component of postoperative pain management needs), a rational and multimodal approach proves necessary to target both peripheral and central pain pathways.

## 4. Perioperative Cognitive Dysfunction in the Elderly

The exact pathophysiology of postoperative delirium (POD) and cognitive dysfunction has not been completely defined nor has all the potential pathways been determined. Both postoperative delirium and cognitive dysfunction have a higher incidence in the elderly surgical population. However, it has been theorized, that by avoiding or reducing centrally acting general anesthesia agents and centrally acting opioids (when possible), RA, along with multimodal analgesia (nonopioids) may optimize perioperative pain management. Therefore, such an anesthetic/analgesic patient-directed and procedure-targeted plan may serve to reduce emergence delirium and postoperative cognitive dysfunction (POCD) in the elderly.

Incorporating general anesthesia has a potential pathogenic factor towards brain toxicity, inflammation, and POCD [7, 8]. Such findings imply that modern general anesthetics being completely reversible may not be entirely correct and that differences along with advantages in perioperative outcome may be evident when using local anesthetic techniques versus reliance on inhalation agents. However, there is not yet a firm correlation between anesthetic technique and long-term pain management improvement or reduced incidence of POCD. Nevertheless, some studies do demonstrate that short-term gains (diagnosed seven days after surgery) have been achieved with RA techniques using local anesthetics compared to more conventional general anesthesia in certain patient populations [9].

Dependence on opioids as the major role for pain management in the elderly can result in a host of well-known AE [10]. Narcotic use has been linked to a disturbed sleep architecture (reduced rapid eye movement and slow wave sleep) that can lead to hyperalgesia. Increases in pain due to hyperalgesia can further worsen sleep and continue to perpetuate the cycle [11]. Following opioid administration, it is established that inadequate sleep can lead to cognitive dysfunction as both adenosine and acetylcholine levels decrease in the basal forebrain. Adenosine and acetycholine levels not only help regulate centrally perceived pain but also play a role to control memory, attention, and wakefulness, all of which are disturbed by opioids. The elderly are particularly sensitive to reductions in these chemical mediators (adenosine and acetycholine) as their baseline reserves are typically already reduced. Therefore, by minimizing reliance on opioids by making perioperative RA the mainstay for pain medicine, AEs could be reduced.

Pain management can be challenging to treat effectively in those suffering from perioperative cognitive changes. Stroke is one possible cause for negative influences on cognition in patients but occurs relatively infrequently [12]. Cognitive disorders can occur in all patients in which mental function diminishes in the early postoperative period and returns to preoperative levels within 7 days after surgery. In addition, CNS dysfunction can be a common finding in elderly patients with some common causes of perioperative cognitive changes including POD and POCD. POD is the most common psychiatric/cognitive dysfunction condition of hospitalized patients. The incidence of POD and POCD may exceed 50% in certain surgical settings (cardiac and orthopedic surgeries) [13, 14]. Incidence of POD and POCD is higher than other postoperative comorbidities such as respiratory failure and myocardial infarction [15], and, in some cases, POD/POCD makes it difficult to treat pain since pain expression(s) cannot be properly assessed. Therefore, effective implementation of RA could reduce the surgical and anesthesia stressors responsible for the transition into POD and POCD.

Older individuals with certain coexisting medical illnesses, patients with preoperative cognitive dysfunction, geriatric patients undergoing certain high-risk surgeries, and surgical candidates of advanced age are at higher risk for development of postoperative cognitive disorders and potential for long-term cognitive dysfunction. Cognitive disorders in high-risk elderly patients occur more frequently than anticipated as is evident in one study in which patients (*N* = 1200) older than 60 years had a 25.8% incidence of cognitive impairment postoperatively at one week and, in some patients (9.9%), if the impairment persisted for 3 months following surgery [16], outcomes ended in a higher mortality rate one year later. Therefore, incorporating RA in a procedure- and patient-specific manner during major surgery in high-risk elderly patients may reduce the incidence of worsening baseline cognitive dysfunction or negative impact towards coexisting disease(s) affecting cognition.

POD and POCD complications can be significant because such adverse outcomes can result in an increased length of hospital stay and medical complications (prolonged acute care hospitalization), unmet postoperative analgesic needs, unplanned discharge to a skilled care facility, and even premature death [17]. Patients with POCD are at an increased risk of death in the first year after surgery [18, 19] and economic impact(s) of delirium is also considerable, adding costs to hospitalization, including billions in additional Medicare charges.

### 4.1. Effects of Cognitive Dysfunction on Perioperative Pain Management

Conditions involving cognitive dysfunction in the elderly are identified in Table 3. Elderly patients often present with age-related changes of their organ systems, and, whether these changes are normal or pathologic, they are to be considered in anesthetic and analgesic management. Perioperative evaluation for major surgery in older patients should be performed as a multidisciplinary team approach to achieve optimal postoperative pain management, recovery, and long-term followup when indicated. In addition, alterations of functional cognitive reserve in the elderly may be reflected as increased susceptibility to POCD, delirium, altered pharmacodynamics, stroke, hyperalgesia, sleep disturbances, and so forth. Therefore, properly selected regional techniques (patient- and procedure-specific) capable of minimizing AE on fragile cognitive impairment for older surgical candidates may yield more optimal short- and long-term outcomes.

Cognitive status often relates directly to a patient's functional ability which can be a determining factor in rehabilitation, pain management, and whether or not a patient is discharged to home or will require a skilled care facility for recovery. Therefore, functional status of elderly surgical patients may be more relevant than their medical morbidity outcomes following surgery. In addition, functional status serves as a strong predictor of mortality as a result of hospitalization [20]. Decreased neurocognitive function yields a decrease in health related quality of life along with adverse financial and social impact for patients, their families, and their care providers [21]. Therefore, if properly implemented and in a timely fashion, regional anesthesia may provide a significantly lessened negative impact upon cognitive functional status in older surgical patients presenting for major surgery.

## 5. Influence of Aging Patients on Regional Anesthesia and Multimodal Pain Therapy

Anesthesia choices using regional techniques can provide targeted pain relief and reduce dependence upon amounts of systemic opioids during the perioperative period. Multimodal nonopioid medication regimens using local anesthetics provide analgesia and reduce reliance on opioids and their many associated AE in the elderly. Optimal postoperative analgesia is achieved when regional analgesics are placed in close proximity to the corresponding dermatome distribution of the surgical site [22–26].

Analgesic agent choices used with regional anesthesia/analgesia (local anesthetics with or without opioids and other adjuncts) can influence patient outcome. Central-neuraxial opioids prove effective in controlling postoperative pain, but only neuraxial local anesthetics (alone) have the ability to attenuate adverse pathophysiological responses that can contribute to perioperative morbidity [27]. Neuraxial local anesthetics are effective through prevention of spinal reflex inhibition of diaphragmatic and gastrointestinal function, suppression of responses to surgical stress, and through blockade of efferent and afferent nerve signals to and from the spinal cord. Perioperative regional techniques in the elderly will influence and control perioperative pathophysiologic events by decreasing respiratory complications, reduce the neuroendocrine stress response, improving effective pain control, promote return of gastrointestinal function, and facilitate earlier patient mobilization. All of the above factors, and many more, play an integral role in management of recuperating patients and can be influenced by compromised organ system reserves of older patients [28, 29].

Despite the lack of conclusive, long-term evidence that regional versus general anesthesia provides improved outcomes, there are studies that can demonstrate improved elderly patient results secondary to reducing organ dysfunction. For example, amelioration of negative pathophysiologic events and improved analgesia with the use of perioperative regional modalities as part of a multimodal pathway for rehabilitation can create fewer stresses on organ function (especially cardiovascular, respiratory, and neurologic). Postoperative regional analgesia as part of a perioperative multimodal approach in patients undergoing abdominal-thoracic esophagectomy can result in a shorter time to patient extubation, earlier return of bowel function, superior analgesia, and earlier fulfillment of discharge criteria from an intensive care unit [30]. Patients participating in a perioperative multimodal pathway following major surgery had a decrease in metabolic and hormonal stress along with improvement in convalescence [31]. Patients undergoing colon resection incorporating epidural analgesia and receiving a multimodal approach to surgical rehabilitation showed a decreased length of hospitalization from 6 to 10 days to a median of 2 days [32]. Therefore, incorporating regional techniques (and utilizing a multimodal anesthetic approach) during the perioperative period may result in accelerated patient recovery for the elderly [33], maximize surgical rehabilitation, aid in attenuation of pathophysiological surgical responses, and reduce the length of hospitalization. Such improvements for older patients may continue to become more obvious when incorporating regional anesthesia in those with aging organ systems, compromised organ reserves, and patients with deleterious effects on organ function secondary to comorbid disease(s). 

### 5.1. Regional Anesthesia and Analgesia and Alterations in the Geriatric Nervous System

Brain sensitivity to anesthetic and analgesic agents increases with age and is unique to each drug. Pharmacokinetic changes that accompany aging and the geriatric nervous system can explain some components of analgesic drug responses seen in the elderly. The mechanisms that define altered brain pharmacodynamics to anesthetics and analgesics in the elderly are unclear at the present, although altered brain kinetics may provide some direction. Age related altered brain sensitivity might result from changes in receptors, signal transduction, and homeostatic mechanisms of the CNS. In addition, alterations of brain phospholipid chemistry associated with changes in second messengers, such as diacylglycerol, are evident [34]. Therefore, if RA can accomplish a reduced impact on baseline or altered brain kinetics, then adverse mechanisms of pharmacology toward brain function could be minimized.

Some major signs and symptoms of an altered nervous system in the elderly include altered reflexes, deteriorations of gait and mobility, altered sleep patterns, impairment of memory and intellect, and decrement of the senses. In older individuals, there is a continual loss of neuronal substance and diminished supply of neurotransmitters leading to reductions in brain reserve. Such reductions increase the likelihood of impaired sensitivity to systemic anesthetic and analgesic medications, symptoms and signs of perioperative neurologic dysfunction, increased risk of POCD, and decreases in functional activities of daily living. These negative attributes of systemic drug administration upon brain reserves in the geriatric patient may be reduced with analgesic modalities that focus on pain management in the periphery (i.e., peripheral nerve blockade).

The autonomic nervous system dictates involuntary physiological functions of the body by way of sympathetic and parasympathetic divisions and this system can experience alterations secondary to the aging process. Aging of the autonomic nervous system is characterized by (a) limited adaptability to stress; (b) net increase in the activation of the sympathetic nervous system; (c) decreased basal activity of parasympathetic nervous system; (d) decreased baroreflex sensitivity; and (e) slowing and weakening of homeostatic functions, all of which may play a role in achieving good perioperative pain management. In addition, there are changes that can occur in the older patient's somatic nervous system and include (a) peripheral nerve deterioration; (b) dysfunction of genes responsible for myelin sheath protein components; and (c) decreased myelinated nerve fiber conduction velocity to identify a few of the changes.

In older individuals, the aging autonomic nervous system has reduced abilities to respond to physiologic changes, stresses, surgery, pain, and anesthesia. Likewise, increases in the sympathetic nervous system activity may be more pronounced and are organ specific with the gastrointestinal (GI) system and skeletal muscle as targets. Neuronal noradrenergic reuptake is reduced in the elderly resulting in an increased sympathetic tone of the heart and an increase in basal adrenal secretions. There is a loss of beat-to-beat heart rate variability during respiration in the elderly due to a reduced respiratory vagal modulation of the resting heart. Decreased baroreflex sensitivity is due to increased arterial stiffness and age-associated alterations of the autonomic nervous system. The autonomic nervous system and its effectors play an important role in responses to hemodynamic challenges. Therefore, any imbalance of homeostatic mechanisms in patients of advanced age can result in clinical compromise (orthostatic hypotension, exercise intolerance, increased upper body sweating, and temperature intolerance) that may be reduced by implementing RA/analgesia.

Age related changes in the peripheral nervous system (PNS) and CNS may affect functional outcomes during the perioperative period and should be considered in patients' preoperative evaluation. Normal aging results in biochemical and anatomical changes of the brain and spinal cord along with qualitative and quantitative effects on the nervous system (Table 4). Therefore, effect of aging on functional reserves of CNS and PNS along with potential surgical and anesthetic ramifications must be considered. Anatomical and biochemical changes of the brain and spinal cord include (a) volume of brain mass, number of synapses, and neurotransmitter concentrations; (b) cerebral electrical and metabolic activity; (c) changes in brain nerve fibers; (d) changes within the spinal cord (cervical spinal cord maintains shape but decreases in size); and (e) modification of the bony spinal canal.

Daily living activities can be dramatically affected by age-related memory decline but are not inevitable. Deficiencies of specific neurotransmitters (related to Parkinson's, Alzheimer's dementia, and other brain disorders) often occur in geriatric patients. Memory deterioration can occur in more than 40% of people older than 60 years of age, and progressive loss of intellectual activity along with mental deterioration (senile dementia) happens in 14% of the population aged greater than 75 years [12]. Changes in neurotransmitter activity and concentrations have also been implicated as a factor influencing anesthetic agent sensitivity. Cerebral metabolic activity is decreased in older subjects and may be a result of decreased neurotransmitter concentrations and synaptic activity. Degenerative changes of myelin sheaths in the CNS may lead to cognitive dysfunction through changes in nerve conduction velocity leading to disruption of normal timing of neuronal circuits. Further contributions to cognitive decline are due to loss of cerebral white matter nerve fibers resulting in decreased connections between neurons. Although these changes have been identified in the aging brain, the mechanisms causing effects on functional activity reserve remain unclear. All of the alterations and changes of the nervous system described above can affect perioperative pain management and should be considered when treating older surgical patients. Therefore, measures focused on reducing such a deleterious impact on nervous system reserves will prove beneficial for the elderly surgical candidate.

### 5.2. Regional Anesthesia & Analgesia and the Cardiovascular System in the Elderly

Patients undergoing various orthopedic procedures under neuraxial blockade for perioperative anesthesia and/or analgesia had a one-third reduction in overall mortality from a recent meta-analysis of randomly controlled trials [35]. Another meta-analysis (*N* = 68,723) on Medicare patients found an association of significantly lower odds ratio of death at both periods of 7 and 30 days when using postoperative epidural analgesia [36]. In yet another meta-analysis (*N* = 2427), it was found that patients who received an epidural (with or without general anesthesia) had a reduced incidence of perioperative myocardial infarction. And, in those instances when a thoracic epidural was maintained for analgesia longer than 24 hours, results showed significantly fewer postoperative myocardial infarctions [23, 37].

Aging of the heart and vascular system in older surgical patients has clinical implications for treatment of postoperative pain management. Morphological and functional changes of the cardiovascular system occur with the aging process (Table 5). There is not scientific evidence to suggest differences in cardiovascular outcome, morbidity, or mortality when using regional modalities or multimodal drug therapy in the elderly [38], but some studies do show a significant benefit that can influence the short-term survival with appropriate application of regional anesthesia [23, 24, 39, 40]. As an example, an epidural combined with general anesthesia for elective abdominal aortic aneurysm repair resulted in a shorter duration of postoperative intubation, reduced incidence of morbidity and mortality, reduced resource utilization, decreased time in the intensive care unit, more optimal postoperative pain relief, and improved overall outcome [39].

Preoperative placement of a continuous epidural in elderly patients for hip fracture surgery (versus a regimen of systemic opioids) has also demonstrated better perioperative pain management and a reduced incidence of adverse cardiac events [40]. In addition, thoracic epidural analgesia could minimize adverse cardiovascular pathophysiology because an effective epidural (analgesia achieved) decreases cardiac sympathetic outflow yielding a favorable balance between myocardial oxygen supply and demand. The statistically proven benefit from regional procedures on the incidence of myocardial ischemia, infarction, or malignant myocardial arrhythmias still remains uncertain. However, there is one study demonstrating that thoracic epidural analgesia does decrease the incidence of postoperative myocardial infarction along with reduced ventricular malignant arrhythmias [23].

Perioperative deleterious cardiovascular events such as congestive heart failure, myocardial infarction, cardiac arrhythmias, and sudden cardiac death can occur with an increased frequency within the first several days of a major surgical intervention [24, 41]. Activation of the sympathetic nervous system (SNS) can result in potentially negative imbalances between myocardial oxygen supply and demand. A host of perioperative stressors including disrupted activities of daily living, anesthesia, surgery, and postoperative pain can activate the SNS of elderly surgical patients to a varying degree. This in turn may predispose older patients with reduced cardiac reserve to myocardial ischemia and infarction.

### 5.3. Regional Anesthesia & Analgesia and the Respiratory System in the Elderly

Armed with an understanding of the multiple respiratory system changes in older patients, clinicians should always titrate analgesic medications carefully and assess patients frequently for any evidence of potential adverse respiratory effects. For example (a) a greater decrease in arterial oxygen tension and/or an increase in carbon dioxide is necessary to increase minute ventilation in the elderly; (b) elderly patients may not adequately increase their minute ventilation to meet respiratory demands induced by hypoxia and hypercarbia under the stress of illness or injury; and (c) furthermore, this effect is exacerbated by the use of opioids, benzodiazepines, and inhalational anesthesia that can promote respiratory depression [42, 43]. Therefore, perioperative risk of respiratory complications among elderly surgical patients may be explained by functional and structural changes within the pulmonary system commonly associated with aging (Table 6).

It remains intuitively reasonable and clinically prudent to consider that older patients could benefit from regional techniques due to minimal sedation requirements, better postoperative pain control, and preservation of pulmonary function. Yet analysis of several studies reveals that choice of anesthetic shows no significant long-term effect on perioperative respiratory morbidity and mortality within any age group. However, by preserving or quickly restoring respiratory function, epidural anesthesia appears to decrease morbidity, reduce length of hospital stay, and lower healthcare costs. In addition, any adverse respiratory effects from inhalational anesthetics and airway manipulation can be avoided. Therefore, the decision to perform regional anesthesia/analgesia must be assessed on a case-by-case basis considering a patient's pulmonary function, type and duration of planned surgery, and anesthesiologist expertise, along with consideration and reflection of the elderly patient's health status and pulmonary reserve.

General anesthesia may reduce functional residual capacity (FRC) by 15–20% with duration of effect that can last 7–10 days following surgery [44]. Following upper abdominal surgical incisions under general anesthesia, a host of potential pulmonary consequences are possible in older surgical patients: (a) vital capacity (VC) can be reduced (25–50%); (b) postoperative pain along with use of systemic opioid analgesics can contribute to a reduction in tidal volume; and (c) an impaired ability to clear secretions and diminished cough reflexes can lead to atelectasis and increased risk of pneumonia. In addition, elderly patients undergoing general anesthesia are predisposed to atelectasis from a combination of age-associated increases in closing volume and reduced FRC. Furthermore, reduced FRC is associated with ventilation-perfusion (V/Q) mismatching, increased alveolar-to-arterial oxygen gradient, and decreased efficiency of gas exchange. However, during neuraxial anesthesia, FRC is often unchanged from baseline.

Studies have shown that older adults undergoing lower extremity orthopedic procedures have fewer respiratory complications when combining epidural plus general anesthesia compared to general with postoperative intravenous morphine analgesia for pain management. Hypoxic events are diminished and measured levels of pulmonary arterial oxygen (PaO_2_) are higher on post-op day 1 when using epidural anesthesia/analgesia as compared to systemic opioids for perioperative pain management [45, 46].

Negative effects on pulmonary function from opioids and inhalation anesthetics predispose older patients to atelectasis, increased risk of hypoxemia and pneumonia, V/Q mismatch, and other postoperative pulmonary challenges [47]. Blunting of hypoxic pulmonary vasoconstriction (HPV) in the elderly during general anesthesia also causes a greater incidence of intraoperative V/Q mismatch and increased alveolar-to-arterial oxygen gradient. Compared to intravenous systemic or epidural opioids, regional anesthesia using local anesthetics for postoperative pain control may provide a greater safety margin for certain older patients and may be more appropriate for postoperative pain relief [48]. Opioids can result in a higher incidence of hypoxic events compared to regional techniques with local anesthetics alone [49]. Epidural local anesthetics compared to systemic opioids for postoperative analgesia result in a reduced incidence, of pulmonary infection, increased PaO_2_ levels, reduced atelectasis and an overall decrease in pulmonary complications [50]. Epidural local anesthetics alone or local anesthetic-opioid mixtures also result in reduced postoperative pulmonary morbidity following major abdominal and thoracic surgery versus intravenous systemic opioids alone [51, 52].

Although some studies appear to demonstrate a clear advantage of regional anesthesia over general anesthesia with systemic opioids, controversy arises due to lack of differentiation and uniformity of epidural analgesic mixtures, site of surgery in conjunction with level of neuraxial blockade and the amount along with type of systemic opioids used. A meta-analysis of 141 clinical trials examining thoracic epidural anesthesia and analgesia versus general anesthesia and postoperative patient controlled opioid analgesia showed a 39% reduction in pneumonia and 60% reduction in pulmonary depression [19]. In another review by Parker et al. a meta-analysis has shown that neuraxial blockade versus patients receiving systemic postoperative opioids may decrease pulmonary complications in hip fracture surgery leading to shortened intensive care unit (ICU) stays and reduced intubation times [53].

### 5.4. Regional Anesthesia & Analgesia and the Endocrine & Immune Systems in the Elderly

There is a corresponding reduction and deterioration of both cellular and humoral aspects of the immune system as human's age. In addition, communicating capability of circulating immune cells and cytokines of the human immune system serves as one of the body's major defense mechanisms. The thymus gland and hormones responsible for mature T-cell modulation are also reduced with aging, leading to a diminished supply of functioning T lymphocytes. By maintaining homeostasis and reducing surgical stress, regional anesthesia may better preserve the immune system, thereby possibly decreasing postoperative infectious complications in older patients.

In an unstressed state, there is minimal alteration of immune functioning in older patients. However, when they encounter surgical stressors, more clinically significant changes in immune function can become evident. Studies reveal that regional techniques can preserve humoral and cellular immune functions in surgical patients [54], whereas administration of general anesthesia may worsen the immunosuppression response that can occur subsequent to surgery. Therefore, by providing older surgical patients with both procedure- and patient-specific regional options, this may more closely mimic an unstressed state for such individuals and preserve an unaltered underlying immune system.

Plasma glucose normalization and improved glucose tolerance with regional techniques can reduce catabolic response to surgery and improve upon gastrointestinal rehabilitation, economy of proteins, and nutritional status of surgical patients [55]. Surgical stress and pain can often produce hyperglycemia and overall catabolism that may predispose patients, especially critically ill patients, to increased mortality and morbidity including: polyneuropathy, increased incidence of opportunistic infections, and multiorgan dysfunction. However, RA may provide more stable physiological parameters during surgery and theoretically prevent or reduce such surgical stress responses [56]. For example, regional techniques may minimize surgical stress by blocking sympathetic and somatic nervous systems from being activated. In addition, epidural blockade can reduce postoperative hyperglycemia and improve glucose tolerance despite plasma insulin concentrations being unchanged [42].

## 6. Anesthesia and Analgesic Techniques: Impact on Aging Organ Systems

Analgesic modalities providing different postoperative analgesic levels may result in a varying incidence of postoperative cognitive dysfunction. There are many theories that RA in the elderly will reduce the incidence of POCD, as well as other negative factors related to morbidity and mortality [22, 40, 41, 46, 57, 58]. Timely implemented, there is a lower incidence of acute postoperative pain, less sleep disruption, and reduced confusion in elderly patients following hip fracture surgery under RA [53]. High delirium risk surgery (ex. femoral neck fracture repair) performed under spinal anesthesia (without perioperative premedication or sedation) has reported no incidence of delirium in elderly patients [59].

Higher degrees of postoperative pain have been associated with an increased incidence of cognitive dysfunction [60], so it would appear that adequate postoperative pain control would decrease the incidence of postoperative cognitive impairment. This concept was demonstrated in a study by Block et al. in which analgesic regimens consisting of regional techniques using local anesthetics were shown to provide superior pain control over systemic opioids [58] and also to reduce systemic adverse effects of opioids that have been associated with the occurrence of POCD [22].

Epidural analgesia has been shown to reduce the incidence of postoperative pulmonary complications that have also been linked to an increased occurrence of POCD [16, 35, 50]. Even with results as described above, there is no statistically conclusive evidence that regional anesthesia and analgesia are associated with a lower incidence of perioperative compromise in the long-term for older patients. One of the problems in evaluating studies surrounding long term outcomes (ex. cognitive preservation) in elderly surgical patients is design flaws and methodological variability contained in the literature. Attempts at interpreting past and current evidence provide conflicting results and, even in the hierarchy of evidence (meta-analysis of randomly controlled trials and large randomized trials), there is lack of conclusive data to demonstrate preservation of organ function beyond the first few hours to a week after surgery when selecting regional techniques [61, 62]. However, other results from meta-analysis demonstrate significant improvement in mortality when neuraxial blockade is used without general anesthesia [63, 64]. Therefore, until absolute predictors and consequences of organ dysfunction are determined, it remains difficult to make absolute and objective recommendations for appropriate treatment and prevention of compromise or decline within aging organ systems.

Perioperative pain has become a costly and significant public health concern due to associated costs of treatment and resources that are consumed due to prolonged hospital stays, medical management of potential pain medicine adverse events (narcotic side effects), patient readmission for treatment of perioperative pain, and so forth. Acute pain should always be expected in postoperative patients. With aging of the US population, more surgeries are being performed annually that translates into a higher absolute number of older surgical patients where pain management continues to pose a challenge for clinicians. As discussed, inadequate pain management can have profound and even long-lasting negative implications (stresses of surgery & postoperative stress, poor surgical outcome, chronic pain development, etc.). Therefore, increasing evidence shows that uncontrolled or inadequately controlled pain (pain of higher intensity and for longer periods) after surgery serves as a risk factor for the development of organ system compromise and that efforts to manage pain in the perioperative period may be very effective in reducing such a burden.

### 6.1. Neuraxial Regional and Anticoagulation Issues in the Elderly

There are a host of well-known advantages in addition to many other potential benefits associated with RA in the elderly surgical patient. However, the incidence of central-neuraxial neurologic compromise resulting from hemorrhagic complications is not completely known. The incidence of neurologic compromise (hemorrhagic injury) cited in the literature is estimated to be less than 1 in 150,000 for epidurals and less than 1 in 220,000 for spinal anesthetics, and recent epidemiologic surveys suggest that the frequency is increasing and may be as high as 1 in 3000 in some patient populations (ex. patients with varying degrees of coagulopathy, both clinical and subclinical). In addition, the risk of clinically significant bleeding increases with advanced age, the presence of an underlying coagulopathy, associated abnormalities of the spinal cord and vertebral column, difficulty during neuraxial needle placement (ex. multiple attempts), and presence of an indwelling neuraxial catheter during sustained prophylactic anticoagulation.

The American Society of Regional Anesthesia and Pain Medicine (ASRA) convened its Third Consensus Conference on Regional Anesthesia and Anticoagulation and developed practice guidelines/recommendations that summarize evidence-based reviews and provide expert opinion in response to patient safety issues surrounding coagulation concerns during RA [65]. Since there are no current laboratory models to base clinical decision-making along with the rarity of neuraxial hematoma (defies performing any prospective randomized trials), the ASRA consensus statements represent the collective experience of recognized experts in the field of neuraxial anesthesia and anticoagulation. Due to understanding of the complexity of the issue, deciding upon choice of the most appropriate perioperative anesthetic management remains crucial. Therefore, the fund of knowledge portrayed by ASRA is based on clinical series, pharmacology, case reports, and hematology literature, but evidence-based outcomes are currently unavailable. An abbreviated version of the ASRA guidelines for regional anesthesia in patients receiving anticoagulation (prophylactic) is provided in Table 7.

### 6.2. Nonopioid Analgesics as a Multimodal Approach to Pain Medicine in the Elderly

Nonpharmacologic methods may also be useful in elderly perioperative pain medicine including heat or ice therapy as well as psychological support. Pain is a complex experience that is influenced not only by the surgical insult, but also by the individual's cognition, culture, experiences, and behavior. Patients can learn coping mechanisms such as relaxation and imagery to decrease muscle tension, reduce emotional distress, and distract from pain [66].

In addition to regional analgesic modalities using local anesthetics, non-opioid multimodal analgesic options will complement such an initiative with reduced reliance upon unimodal therapy with opioids. Adjuvant drugs such as antidepressants, topically applied single- or multiagent analgesics, corticosteroids, anticonvulsants, skeletal muscle relaxants, and alpha 2 agonists were agents developed with clinical indications for treatments other than for pain, but they have also been discovered to provide analgesia. They should be considered to contribute and improve regional perioperative pain management, reduce dependence on opioids, and limit potential AE from opioids in the elderly. Many of the adjunct analgesics (non-opioid) can be recommended when toxic limits of opioid analgesics are approached or when the therapeutic benefit of a primary analgesic appears to have plateaued. In addition, these medications can be used in the presence of other nonpainful disabling complaints of the elderly such as depression, anxiety, insomnia, and fatigue [67].

Nonsteroidal anti-inflammatory drugs (NSAID) are the most widely prescribed and used medication in the US. NSAID administration is a common non-opioid drug alternative and has been included in multimodal anesthesia/analgesia practice guidelines for acute pain management in the perioperative setting [68]. McDaid et al. demonstrated that NSAID reduce morphine requirements in patient controlled analgesia following major surgery along with a concomitant reduction in morphine-related nausea, vomiting, and sedation [69]. Therefore, perioperative NSAID administration can provide pharmacological action (antipyretic and anti-inflammatory activity) that when used in combination with regional anesthesia may enhance patient satisfaction and quality of their recovery.

Several other adjuvants can provide beneficial value to regional anesthesia/analgesia in the elderly surgical patient. Acetaminophen (non-NSAID analgesic) should be considered as a management therapy for pain. It is ideal for postsurgical therapy because of its platelet sparing properties (recent availability of an intravenous formulation) and reduced concerns during neuraxial and RA administration (already used in multimodal analgesic regimens in several surgical institutions). Incorporation of antidepressant medications into perioperative pain management also has advantages that compliment regional techniques used in the elderly. In addition to their value as an analgesic, antidepressants improve a patient's sense of well-being, reduces fatigue, and do not disrupt the normal sleep cycle. Certain antidepressants (treatment of neuropathic pain characterized by damage/dysfunction of CNS and/or PNS) have become popular as a non-opioid medication in the treatment of postoperative pain as a multimodal component in combination with regional anesthesia. Anxiolytics can be included in a multimodal perioperative pain management regimen. Pain can cause anxiety and, in turn, anxiety often exacerbates the perception of pain. Therefore, anxiolytics may provide a mechanism to break this cycle and indirectly help to relieve pain in combination with regional anesthesia/analgesia.

Other commonly used drugs for the treatment of pain (neuropathic) included in multimodal therapy for acute pain management are anticonvulsant medications. Skeletal muscle relaxants, another adjunct in pain management regimens, have multiple modes of action. The comparative efficacy of these drugs is not well known in the elderly however, evidence from clinical trials is limited and a distinction that needs to be understood is that skeletal muscle relaxants consist of antispasticity and antispasmodic agents. Therefore, skeletal muscle relaxant choice should be based on indication, patient tolerability, and potential AE profile when used in combination with regional anesthesia/analgesia.

## 7. Conclusion

Perioperative pain management in older patients can often be associated with specific complicating elements surrounding perioperative events, especially during major surgery. Geriatric patients typically suffer from a greater number of comorbid diseases, have lower organ function reserve, and demonstrate altered physiologic and pharmacologic reactions to analgesic medications. There are also challenges in pain management and assessment that remain unique in elderly patients. For example, it may require more time for older patients to understand the pain scale due to barriers such as vision, hearing, or cognitive defects. In addition, elderly patients also tend to underreport pain and effective perioperative pain management requires careful assessment of pain and sensible dosing of analgesics along with implementation of regional techniques. It is also crucial to be aware of potential AEs secondary to analgesic options and to keep a close watch for their development, but a focus on reducing or eliminating such events and patient care compromise will yield better surgical outcomes for older patients.

Perioperative regional anesthesia/analgesia as part of multimodal drug therapy may prove to be the most effective approach to perioperative pain management in the elderly, cognitively impaired patients with comorbid disease, and so forth, with the least amount of physiological compromise. Regional anesthesia may benefit older patients by reducing postoperative neurological, pulmonary, cardiac, and endocrine complications and potentially improve upon immediate postoperative pain control, but it has yet to be conclusively proven to improve long-term morbidity and surgical outcome. Multimodal drug therapy incorporating regional options provides a potentially advantageous choice toward perioperative pain management that utilizes a variety of analgesics in order to minimize dosages and reliance of any single agent while maximizing analgesic effect in the older surgical patient population.

Therefore, it remains necessary to implement carefully thoughtout perioperative pain medicine therapies and to continue the search for development of new methods of pain management for all patients, especially the growing elderly surgical population. In addition, if healthcare services are to demonstrate that the many reports of inadequate perioperative pain management (sometimes necessitating readmission to the hospital [70]) are becoming less problematic for the elderly when using regional techniques, then perioperative pain medicine needs to continue to produce randomized, prospective studies that can show conclusive evidence of advantages for the geriatric patient population.

## Figures and Tables

**Table 1:** Regional anesthesia.

Types of upper extremity blockade	Interscalene
	Supraclavicular
	Infraclavicular
	Axillary
	Intravenous regional

Types of lower extremity blockade	Lumbar plexus
	Sciatic
	Femoral
	Lateral femoral cutaneous
	Obturator
	Popliteal and saphenous
	Ankle

Types of head and neck blockade	Cervical plexus
	Stellate
	Occipital
	Maxillary
	Mandible
	Retrobulbar

Types of truncal blockade	Intercostal
	Interpleural
	Paravertebral
	Transversus abdominis plane

Neuraxial	Spinal
	Epidural
	Caudal

**Table 2:** 

Benefits of regional anesthesia	
(i) Improve acute perioperative pain management	
(ii) Reduced opioid use along with reduced incidence of adverse events	
(iii) Can obtain skeletal muscle relaxation, thus limiting need and risks of IV muscle relaxants	
(iv) Option to maintain patient consciousness	
(v) Continued presence of protective upper airway reflexes	
(vi) An isolated regional modality will have minimal effect on pulmonary or cardiac disease	

**Table 3:** 

Definitions of altered mental status	
Condition	Definition
Postoperative cognitive dysfunction (POCD)	Persistent deterioration of cognitive performance after surgery most often resolves within 3 months but can last longer

Delirium	Decline in mental status, reduced awareness, cognitive and psychomotor dysfunction, disorientation, and memory impairment

Altered pharmacodynamics	Increased effect of drug on the body; for example, exaggerated respiratory and cardiovascular depression from narcotics

Stroke	Decreased blood supply to the brain due to ischemia or hemorrhage. There is an increased risk in the elderly, because of autonomic dysfunction, decreased baroreceptor sensitivity, and loss of vascular elasticity

Hyperalgesia	Paradoxical increase in pain intensity, new pain complaints, and altered pain characteristics while receiving treatment with opioids

**Table 4:** 

Changes in the aging central nervous system	
(i) Decreased synaptic transmission	
(ii) Diffuse slowing on EEG	
(iii) Decreased oxygen and glucose consumption	
(iv) Loss of neuronal substance	
(v) Decreased production of neurotransmitters	
(vi) Decrease in cytoplasmic protein synthesis	
(vii) Decreased myelin glycoproteins	

**Table 5:** Cardiovascular system changes in the elderly.

(i) Reduced exercise tolerance	
(ii) Loss of vascular elasticity (e.g., LVH and hypertension)	
(iii) Chronic blood pressure elevation and decreased baroreceptor sensitivity	
(iv) Increase in coronary arteriosclerosis	
(v) Valvular heart disease	

**Table 6:** Pulmonary system changes in the elderly.

(i) Decreased chest wall compliance due to changes in muscle and chest wall joints	
(ii) Increased lung compliance due to loss in parenchymal elasticity	
(iii) Decreased lung parenchymal surface area leading to decreased efficiency of alveolar gas exchange due to increase in dead space and shunt	
(iv) Decrease FEV1, decreased TV, and increased RR	
(v) Hypotonia of pharyngeal muscles leading to upper airway obstruction	
(vii) Decreased responsiveness to hypercapnia and hypoxia	
(viii) Increased work of breathing	

**Table 7:** ASRA guidelines for RA in the patient receiving anticoagulation therapy.

Anticoagulation	Dosage	Recommendations
Unfractionated heparin	SC BID	No contraindications
	SC TID	No recommendations—unknown risk
	IV loading dose	Delay administration >1 hour after needle placement or catheter removal
		Remove catheter 2–4 hours after last dose
LMWH	SC daily	Needle placement or catheter removal 10–12 hours after last dose
		Delay dosing 2 hours after removal of catheter
	SC BID	Needle placement or catheter removal 24 hours after last dose
		Delay dosing 2 hours after removal of catheter
Warfarin		Stop 4-5 days prior to neuraxial technique, INR < 1.5 prior to needle placement or catheter removal
NSAID		No contraindications

GPIIB/IIIA antagonists		
Ticlopidine		Delay 14 days
Clopidogrel		Delay 7 days
Herbal therapy		No contraindications when used independently
